# Dr. G. M. Phadke: The first president of the Association of Genitourologic surgeons

**DOI:** 10.4103/0970-1591.38594

**Published:** 2008

**Authors:** Anita Patel

**Affiliations:** Consultant Urologist, Mumbai, India

**Figure:**
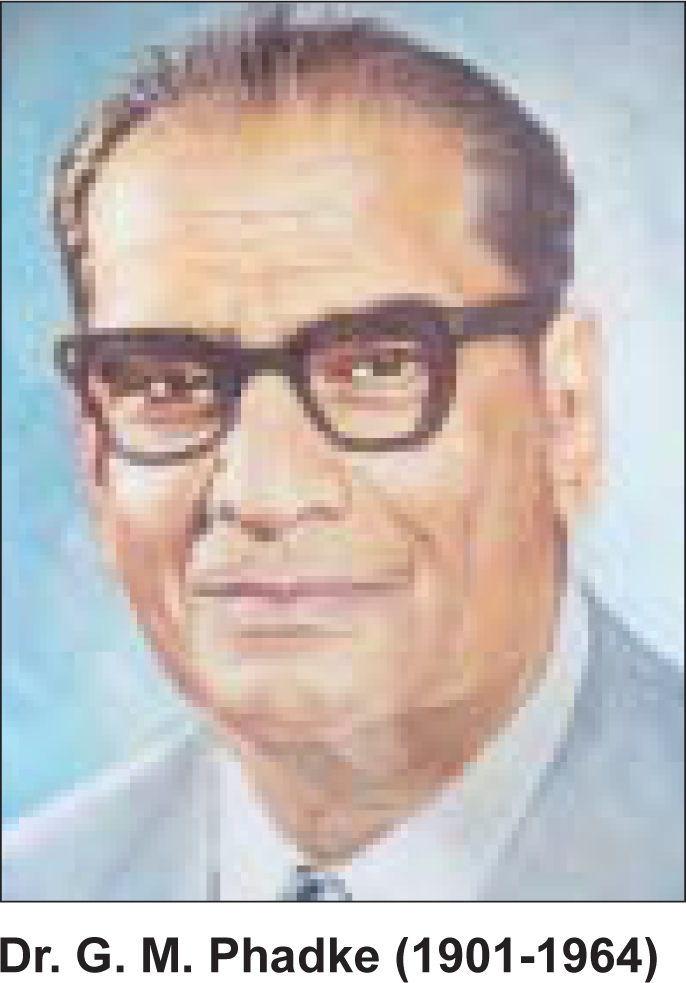
Dr. G. M. Phadke (1901-1964)

In 2007, the Urological Society of India [USI] completes 16 years of its existence as an independent society. However, it was conceptualized many years before and the man responsible for this was none other than Dr. G. M. Phadke, the founder President of the USI. The seeds of the Association of Genitourologic (GU) Surgeons were sown during a meeting of the Association of the Surgeons of India [ASI]. Along with Dr. H. S. Bhat, Dr Colabawalla and Dr. S. Mansingh, Dr. G. M. Phadke started this association and became its founder President.

The first sectional meeting of this association took place in Christian Medical College (CMC), Vellore in 1963, with Dr. H. S. Bhat as the organizing Chairman and Dr. Ashok Bhajekar as the organizing secretary. Needless to say, this was just the beginning of what is now known as the Urological Society of India (USI).

Dr. G. M. Phadke was born in 1901 into a prominent zamindar family in Khandesh. He was a bright and ambitious student and had always dreamed of becoming a doctor. After completing his matriculation at the age of 19, he left for the UK to pursue higher education.

The next 12 years that he spent in the UK were momentous for him as they moulded his character and gave direction to his life. Dr. Phadke graduated from the prestigious University College Hospital, London and obtained a fellowship of the Royal College of Surgeons of England. Besides pursuing his medical studies, he also excelled in golf, skiing and ballroom dancing. After gaining considerable surgical experience, Dr. Phadke returned to India at the advice of his mentor, the famous neurosurgeon, Sir Wilfred Trotter.

On his return from the UK in 1933, Dr. Phadke joined King Edward Memorial (KEM) Hospital, Mumbai as Assistant Surgeon. He got married in 1933 and Ajit, his son (now an eminent urologist himself) was born in 1935. He started the Colony Nursing Home in the heart of Matunga in 1935 in collaboration with Dr. V. N. Shirodkar. With his humane approach and exceptional surgical skills, Dr. G. M. Phadke soon became the most sought-after surgeon of his time. His fame, reputation and loyalty to his profession earned him patients nationwide including many prominent personalities.

His career took an interesting turn when a senior venereologist colleague, Dr. A. P. Pillay started referring a lot of men with “infertility” and obstructive azoospermia to him for further treatment. In the twentieth century, smallpox was a common disease in India. Dr. G. M. Phadke and his colleague, Dr. A. M. Phadke, were the first to report that smallpox was the most common etiological factor for obstructive azoospermia in India. They also showed that vasoepididymal anastomosis was successful in improving sperm counts.

Around the same time, the association of Planned Parenthood [APP] from New York was trying to gain a foothold in India. In collaboration with Dr. G. M. Phadke and Dr. V. N. Shirodkar, APP started the Family Planning Association [FPA]. Interestingly, Dr. Phadke was also instrumental in advocating vasectomy as a highly effective and harmless family planning operation. He also busted several myths about vasectomy and convincingly demonstrated its successful reversibility (VVA). The Films' Division of India even made a documentary film entitled, “Role of vasectomy in the family planning campaign in India” with Dr. G. M. Phadke's help. Although andrology was his initial interest, urology soon became equally important. In the early 50's, Dr. Phadke went back to England and learned retropubic prostatectomy from the famous Dr. Terrence Millins. He was also very fond of performing Johanson urethroplasty for treating urethral strictures.

He joined Bombay Hospital in 1950 and continued to offer his dedicated services until 1964. He was very fond of teaching small groups of medical students. His simple words and tricks of wisdom made his bedside clinics a big hit in KEM Hospital. With his love for teaching, he made a mark at the national level. Attending the annual conference of the ASI was an annual pilgrimage for him. He was elected as the President of the ASI in 1957 and later was responsible for starting a separate section on urology.

Apart from urology, Dr. G. M. Phadke was an avid fan of music and in particular, enjoyed listening to Marathi Natya Sangeet. He was one of the trustees of Marathi Sahitya Sangh and enjoyed watching Marathi plays. In addition to his professional excellence, he was also a great humanitarian and never charged any money for his professional services from teachers or performing artistes. Although his professional commitments did not allow him to spend much time at home, he was very particular about having at least one meal every day with his entire family in attendance including his ageing mother.

Dr. G. M. Phadke passed away following a massive myocardial infarction in 1964. Apart from his innumerable grateful patients and students, the USI also owes a lot to this legendary figure and salutes his spirit. The USI has instituted a traveling fellowship and an oration in memory of Dr. G. M. Phadke.

